# Controlling the Kinetics of an Enzymatic Reaction through Enzyme or Substrate Confinement into Lipid Mesophases with Tunable Structural Parameters

**DOI:** 10.3390/ijms21145116

**Published:** 2020-07-20

**Authors:** Marco Mendozza, Arianna Balestri, Costanza Montis, Debora Berti

**Affiliations:** Department of Chemistry “Ugo Schiff” and CSGI, Via della Lastruccia 3-13, 50019 Florence, Italy; marco.mendozza@unifi.it (M.M.); arianna.balestri@unifi.it (A.B.); debora.berti@unifi.it (D.B.)

**Keywords:** liquid crystalline mesophases, phytantriol, drug delivery, controlled release, enzymatic kinetics, alkaline phosphatase, confined diffusion

## Abstract

Lipid liquid crystalline mesophases, resulting from the self-assembly of polymorphic lipids in water, have been widely explored as biocompatible drug delivery systems. In this respect, non-lamellar structures are particularly attractive: they are characterized by complex 3D architectures, with the coexistence of hydrophobic and hydrophilic regions that can conveniently host drugs of different polarities. The fine tunability of the structural parameters is nontrivial, but of paramount relevance, in order to control the diffusive properties of encapsulated active principles and, ultimately, their pharmacokinetics and release. In this work, we investigate the reaction kinetics of p-nitrophenyl phosphate conversion into p-nitrophenol, catalysed by the enzyme Alkaline Phosphatase, upon alternative confinement of the substrate and of the enzyme into liquid crystalline mesophases of phytantriol/H_2_O containing variable amounts of an additive, sucrose stearate, able to swell the mesophase. A structural investigation through Small-Angle X-ray Scattering, revealed the possibility to finely control the structure/size of the mesophases with the amount of the included additive. A UV–vis spectroscopy study highlighted that the enzymatic reaction kinetics could be controlled by tuning the structural parameters of the mesophase, opening new perspectives for the exploitation of non-lamellar mesophases for confinement and controlled release of therapeutics.

## 1. Introduction

Polymorphic lipids have been widely studied over the years, for their unique self-assembly properties: in water, they can form lyotropic liquid crystals with long-range order promoted by hydrophobic forces and H-bonds [1], with different arrangements and a complex phase diagram highly dependent on temperature, pressure [2], water content and ionic strength [3,4,5,6]. 

Glycerol monooleate (GMO, a glycerol monoester) and phytantriol (Phyt, a terpenoid polyalcohol) are among the most studied polymorphic amphiphiles and are characterized by a particularly rich phase diagram. Depending on the experimental conditions, they assemble into hexagonal mesophases (consisting of lipid monolayered cylinders arranged into a hexagonal network), lamellar mesophases (consisting of lipid bilayers arranged in stacked infinite plans divided by water regions), or inverse bicontinuous cubic mesophases (consisting of a lipid bilayer wrapped on a periodic minimal surface [7]). 

Cubic mesophases are the most studied structures, both for GMO and for Phyt, being the thermodynamically stable phases in excess water at room temperature; they can exhibit different arrangements, namely Schwarz diamond, primitive and Schoen gyroid, characterized by different infinite periodical minimal surfaces (IPMS) with spatial groups Pn3m, Im3m and Ia3d, respectively, with the hydration level and curvature of the phase increasing from gyroid, to diamond, to primitive structure [7]. While gyroid and diamond phase are the thermodynamically stable phases with excess water, in different experimental conditions, the primitive cubic phase arrangement is generally observed in the presence of additives [8] and polymers [9] swelling the water channels. 

Due to their unique structure, characterized by the coexistence of extended hydrophobic and hydrophilic regions arranged in a continuous 3D complex architecture, cubic mesophases, in their disperse form, i.e., cubosomes, have been widely studied as possible alternatives to the most common lamellar phases (in particular in their dispersed form, as liposomes) for the development of vectors for drugs of different size and polarity for biomedical applications [6,10,11,12]. They have been formulated for local and systemic administration, in different routes [13]. Compared to liposomes, cubic nanoparticles are characterized by a reduced internal aqueous volume to host hydrophilic drugs; however, their extended membrane surface is particularly suitable for loading of membrane proteins and small drug molecules, as well as hydrophobic molecules [14]. For instance, they have been employed to transport small hydrophobic molecules as quercetin [15,16] and camptothecin [17] and photosensitizers [18], which can be included in the extended hydrophobic regions of cubic mesophases, or hydrophilic or amphiphilic macromolecules and proteins [10,19,20], which can be efficiently hosted and retained in the peculiar structure of the aqueous regions, comprising cages and necks. in addition, cubic mesophases have been successfully loaded with hydrophobic or hydrophilic superparamagnetic iron oxide nanoparticles, to build-up smart drug delivery systems, able to release the encapsulated drugs in a spatially and temporally controlled manner [21,22]. Finally, it has been shown that cubic mesophases have a strong ability to interact with phospholipid cell membranes, making them particularly interesting for the intracellular delivery of drugs.

Despite these promising features, one drawback in the application of cubic mesophases as drug delivery systems is the fact that, once temperature and pressure are defined, their structural parameters in excess water are poorly tunable, which might result in a limited control of the pharmacokinetic properties (i.e., the release profile upon loading) of drugs encapsulated in the cubic mesophases. In recent years the effects of additives on the phase diagram of cubic mesophases has started to be explored as a possible strategy to tune the arrangement of the lipid scaffold and to widen the dimension ranges of hydrophobic and hydrophilic domains, in order to better host and retain hydrophobic and hydrophilic drugs of different sizes, and/or tune the physical-chemical features of the liquid crystalline mesophases. For instance, fatty acids [23,24], phospholipids [25,26], photo-switchable molecules [27,28,29] or also nanoparticles, such as iron oxide [22,30], gold [31] and quantum-dots [32], have been shown to effectively modify the phase diagram of cubic mesophases, both in terms of lattice parameters and in terms of shift of the phase borders [33]. Recently, Mezzenga et al. have reported on the swelling of monolinolein Pn3m modified by sugar esters [8,20]; the additive promotes a transition to Im3m cubic mesophase, with water nanochannels about 2.5 times larger than in pure lipid/water systems. This variation, as highlighted in several studies, can modify the confinement level of the encapsulated drug, ultimately controlling its release profile [8,34,35]. In a recent work by Assenza et al. [36] it has been hypothesized that for relatively large channels the diffusion coefficient of an encapsulated molecule can be related to the average of the Gaussian curvature; conversely, in the case of narrow channels, a symmetry-dependent diffusion reduction is observed [36]. In general, the structure/symmetry of the mesophase, the size of the lattice parameters and of the nanochannels and, finally, the size and polarity of the drugs to be included in the mesophase will all affect the release profile of the drug in the surrounding environment. 

In this contribution we address this issue, i.e., the relationship between the structural control over self-assembled cubic mesophases with determined and variable structural parameters, and their retention/release ability with different drugs. In particular, here we investigate Phyt/water system in the temperature range 25–50 °C loaded with variable amounts of the additive sucrose stearate (SS), a sugar ester able to swell the water channels. Through small-angle X-ray scattering (SAXS) we characterized the phase diagram of Phyt/SS/H_2_O, highlighting the possibility to achieve a fine structural control over the mesophases’ dimensions. In addition, we included in the aqueous channels of the lipid mesophases with different lattice parameters, two model hydrophilic guest molecules, differing in size and function: an enzyme (alkaline phosphatase, AP, M_W_ = 140 kDa) and its substrate, i.e., p-nitrophenyl phosphate, a small molecule with M_W_ = 263.05 Da. The purpose of this work was twofold: i) verify how the active molecules diffuse and are released from the different lipid mesophases characterized by different lattice parameters and ii) evaluate the impact of confinement on the overall enzymatic reaction.

## 2. Results and Discussion

### 2.1. Structure of Phytantriol/Sucrose Stearate Lipid Mesophases

To determine the impact of sucrose stearate (SS) on the arrangement of Phyt mesophases in excess water, we characterized the structure of Phyt/water binary systems, upon inclusion of increasing amounts of SS, at different temperatures. Phyt, in excess water, is characterized by a well-known phase diagram [37], i.e., it arranges in a Pn3m cubic structure until the temperature reaches 45–50 °C, over which it undergoes a phase transition to a hexagonal phase. 

**Figure 1:**
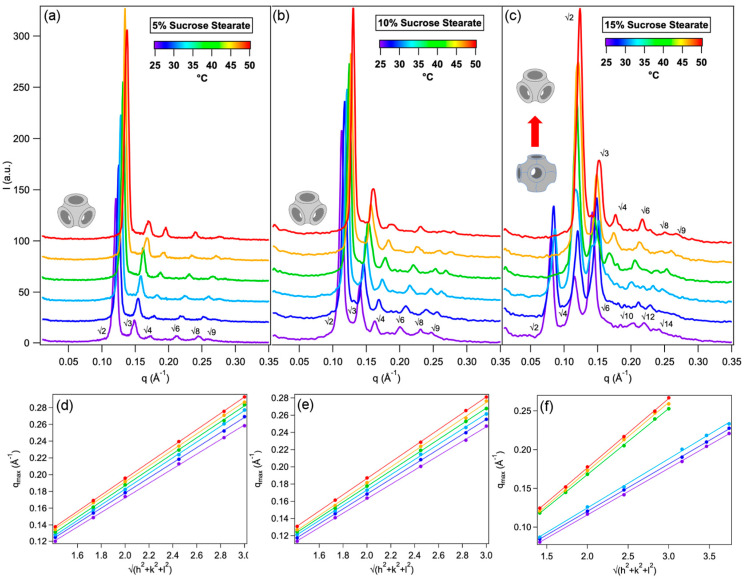
Structure of Phyt mesophases doped with SS. SAXS profiles of Phyt mesophases containing 5% (**a**), 10% (**b**) and 15% (**c**) *w*/*w* SS, in excess water, measured at 25, 30, 35, 40, 45, 50 °C. (**d**), (**e**) and (**f**) show the linear plots of the Bragg peaks q-values vs. h, k, l indices, to estimate the lattice parameters of the mesophases with 5%, 10% and 15% SS, respectively.

Figure 1 shows representative small angle X-ray scattering (SAXS) curves of Phyt cubic mesophases with increasing concentrations of SS, namely 5% (Figure 1a), 10% (Figure 1b) and 15% (Figure 1c) measured at 25, 30, 35, 40, 45, 50 °C. The lattice parameters, reported in Table 1, display a well-known trend with increasing temperature, that is, a progressive decrease of the lattice parameter, associated to an overall shrinkage of the lipid mesophase; this effect is related to the conformation of the amphiphile chains splaying away each other, therefore increasing the molecule hydrophobic molecular portion. In terms of curvature of the lipid membrane, increasing the temperature results in driving a more negative spontaneous curvature of the leaflets, leading to an overall shrinking of the lipid mesophase [7,38]. 

For the lowest and intermediate concentration of SS (5% and 10% *w*/*w*), the Pn3m structure of the mesophase is retained in the whole temperature range investigated (25–50 °C); however, for each temperature monitored, the lattice parameters of the liquid-crystalline phase are progressively increased by the addition of SS. This effect can be attributed to a localized perturbation of the mesophase due to the presence the additive: the sugar esters are localized at the water-lipid interface, with the hydrophobic portion inserted in the lipid bilayer and the highly hydrophilic sugar portion protruding in the water channels, enhancing the amount of water molecules in the nanochannels. This localized perturbation determines a decrease (in absolute value) of the curvature of the membrane, overall promoting a swelling of the lipid mesophase. This effect is much more pronounced for the sample where 15% *w*/*w* SS is present: in this case, at 25 °C the phase arrangement is Im3m, characterized by a lower curvature of the membrane with respect to the Pn3m. We notice that increasing amounts of SS produce an opposite effect with respect to temperature increase (i.e., the addition of SS decreases the local curvature, while the temperature increase enhances it). Remarkably, at 40 °C, the sample Phyt/SS(15% *w*/*w*)/H_2_O undergoes a transition from Im3m to Pn3m, i.e., the thermodynamically stable structure for Phyt/H_2_O in the absence of additives at room temperature. Therefore, for this sample and temperature range, the swelling-deswelling effects (SS inclusion and temperature increase) are perfectly counterbalanced and the lattice parameter is similar to that observed for the Phyt/H_2_O binary system at 25 °C (see Table 1). 

From these results it appears that, by playing with temperature and additive amount, it is possible to finely tune the structural parameters of a Phyt/H_2_O mesophase. To this aim, we determined the size of the nanochannels from the lattice parameters (reported in Table 1) through Equation (1) for a cubic phase:

(1) $$r_{w}=\sqrt{\left(-A_{0}/2\pi \chi \right)}d-l_{c}$$

and Equation (2) for a hexagonal phase:

(2) $$r_{w}=\frac{0.525d-l_{c}}{0.994}$$

where *r_w_* is the radius of the water channel, *A*_0_ and *χ* constants related to the topology of the mesophases (for a Pn3m *A*_0_ = 1.919 and *χ* = −2 while for an Im3m *A*_0_ = 2.345 and *χ* = −4), *d* is the lattice parameter, experimentally determined from the SAXS curves and the lipid bilayer thickness is considered equal to 2*l_c_* = 2.84 nm both for pure Phyt/water system and for the systems augmented with SS.

The values of the diameters of water nanochannels determined through the previously reported equations reflect the trend of the lattice parameters of the mesophase: for instance, at 25 °C the diameter of water nanochannels are 2.2 nm for neat Phyt/H_2_O system, and it progressively increases to 2.8 nm for 5% *w*/*w* SS, to 3.0 nm for 10% *w*/*w* SS and, finally, to 3.6 nm for 15% *w*/*w* SS. On the other hand, for each mesophase the temperature increase determines a progressive shrinkage of the water channels, determining a progressive water release from the mesophase. This effect is more pronounced in case of the system containing 15% *w*/*w* SS, where between 35 and 40 °C a Im3m to Pn3m phase transition occurs, which is accompanied by a burst decrease of the water content of the mesophase. 

In summary, SAXS investigation highlights that a library of liquid crystalline mesophases of cubic structure is formed, characterized by variable and controlled size of the nanochannels. In the following paragraph we will report on the characterization of the enzymatic reaction involving the enzyme alkaline phosphatase and its substrate p-nitrophenyl phosphate (Section 2.2), which will then be included in the mesophase (Section 2.3 and Section 2.4), to monitor the effect of confinement on the kinetics of the enzymatic reaction.

### 2.2. Alkaline Phosphatase Enzymatic Reaction in Aqueous Solution

**Figure 2:**
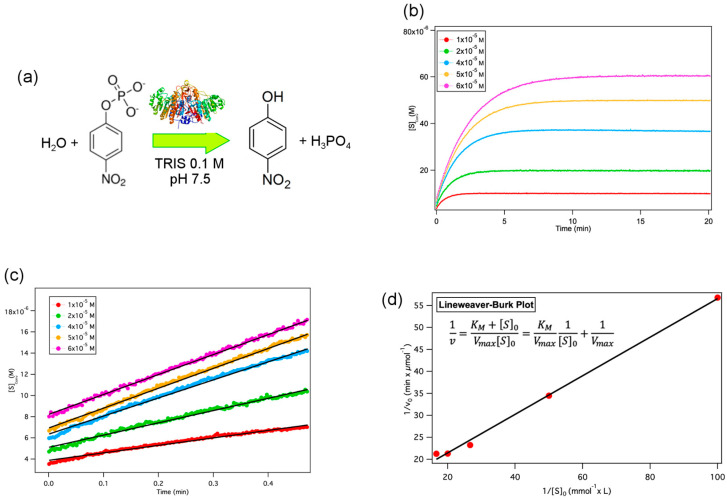
Reaction kinetics in solution. (**a**) Sketch of the AP-catalysed reaction of p-nitrophenyl phosphate conversion into p-nitrophenol; (**b**,**c**) reaction kinetics followed through UV–vis spectroscopy at 400 nm, varying the concentration of substrate (1 × 10^−5^ M (red), 2 × 10^−5^ M (green), 4 × 10^−5^ M (cyan), 5 × 10^−5^ M (yellow) and 6 × 10^−5^ M (purple) in the presence of a fixed amount of enzyme (0.2 U/mL); (**b**) complete conversion kinetics (0–20 min) (**c**) initial conversion kinetics (0.0–0.5 min) analysed with a linear fit (black bold lines) to determine the initial reaction rate; (**d**) Lineweaver–Burk plot of the inverse initial reaction rate vs. the inverse substrate concentration to extract the Michaelis–Menten constant and the maximum velocity; the error bars in the graph are smaller than the markers’ size.

Alkaline phosphatase (AP) catalyses the conversion of the colourless substrate p-nitrophenyl phosphate to a yellow-green product (p-nitrophenol) in TRIS buffer, pH 7.5 (see Figure 2a). The enzymatic reaction and the progressive formation of the reaction product can be therefore conveniently followed through UV–vis spectroscopy, observing the absorbance increase at 400 nm. 

Figure 2b shows the reaction kinetics profiles at different substrate concentrations (1 × 10^−5^, 2 × 10^−5^, 4 × 10^−5^, 5 × 10^−5^, 6 × 10^−5^ M), monitored in solution, in the presence of 0.2 U/mL of AP. As clearly highlighted from Figure 2b, p-nitrophenol absorbance at 400 nm reaches a plateau after 5–15 min, depending on the concentration of the substrate, proving that the reaction is complete in this timeframe. 

Figure 2c reports p-nitrophenol absorbance increase in the first 30 s, showing that the initial reaction rate depends critically on the substrate concentration. To determine the Michaelis–Menten constant, *K_M_* and the maximum reaction rate, *V_max_*, we used the linear Lineweaver–Burk plot (Equation (3)):

(3) $$\frac{1}{v}=\frac{K_{M}+{\left[S\right]}_{0}}{V_{max}\;{\left[S\right]}_{0}}=\frac{K_{M}}{V_{max}}\frac{1}{\;{\left[S\right]}_{0}}+\frac{1}{V_{max}}$$

where *v* is the initial reaction rate, [*S*]_0_ the substrate concentration; from the plot reported in Figure 2d, the evaluated Michaelis–Menten constant *K_M_* and *V_max_* values are *K_M_* = 0.034 mM and *V_max_* = 7.9 × 10^−2^ μmol of product/min, respectively. These results substantially agree with the literature values [39], taking into account the different experimental conditions here adopted, i.e., 25 °C instead of 37 °C, neutral instead of alkaline pH, lower enzyme/substrate ratio.

### 2.3. Alkaline Phosphatase Substrate Inclusion in Lipid Mesophases

**Figure 3:**
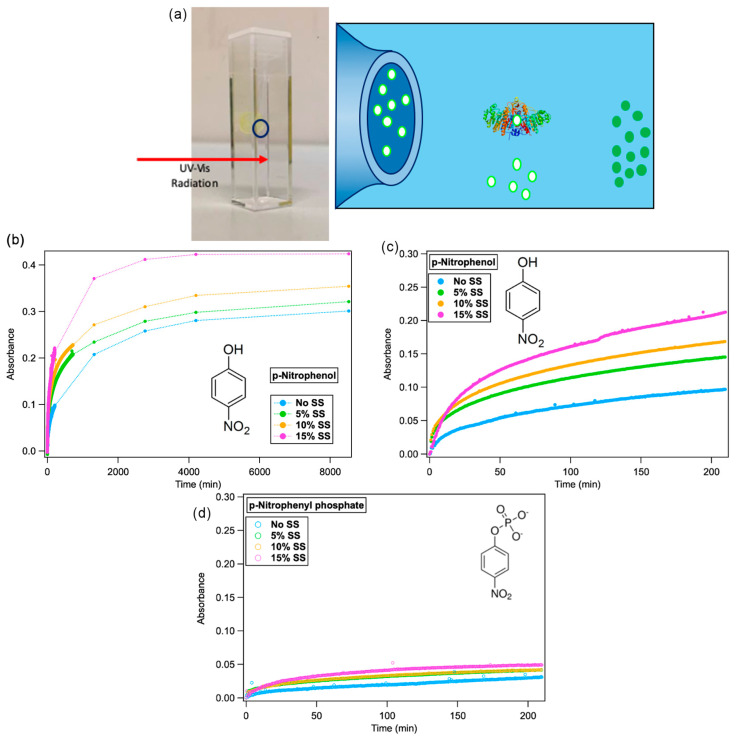
Reaction kinetics with the substrate confined in liquid crystalline mesophases. (**a**) Sketch of the experiment of AP-catalysed reaction with the substrate confined in the cubic mesophase; (**b**,**c**) UV–vis absorbance of p-nitrophenol in solution, recorded at 400 nm for 4 h (**b**) and six days (**c**); (**d**) UV–vis absorbance of p-nitrophenylphosphate in solution, recorder at 310 nm for 4 h. The different curves are related to experiments performed with the substrate confined in the different mesophases: No SS (cyan markers), 5% SS (green markers), 10% SS (orange markers) and 15% (purple markers).

We then addressed the effect of confining the substrate in the mesophases aqueous channels of varying sizes. The inclusion of the substrate in the liquid crystalline mesophases did not significantly affect the structure of the mesophases, as highlighted from SAXS (see SI, Figure S1 for details). The experimental set-up can be briefly described as follows: Phyt or Phyt/SS was hydrated with appropriate amounts of a p-nitrophenyl phosphate solution and placed in a UV–vis cuvette containing 2.5 mL of a 0.2 U/mL AP aqueous solution (TRIS pH 7.5) and the absorption increase monitored for six days. This setup allowed monitoring the absorbance in the external medium, excluding the mesophase region. The concentration of substrate into the mesophases was chosen to reach a theoretical absorbance value A = 1 (concentration of the product 6 × 10^−5^ M). Therefore, the initial absorbances of substrate and product are, for this configuration, zero. Figure 3c,d display the UV–vis spectra measured for the enzymatic reaction over the first 4 h. In particular, the release of p-nitrophenyl phosphate from the cubic phase of Phyt containing different amounts of SS (the absorbance at 310 nm is monitored over time, Figure 3d), and its conversion into p-nitrophenol by the AP enzyme dispersed in the surrounding TRIS buffer solution (the absorbance at 400 nm is monitored over time, Figure 3b,c).

Figure 3c,d displays the UV–vis spectra measured for the enzymatic reaction over the first 4 h. In particular, the release of p-nitrophenyl phosphate from the cubic phase of Phyt containing different amounts of SS (the absorbance at 310 nm is monitored over time, Figure 3d), and its conversion into p-nitrophenol by the AP enzyme dispersed in the surrounding TRIS buffer solution (the absorbance at 400 nm is monitored over time, Figure 3b,c).

To observe the formation of products, two different pathways for substrate/enzyme contact are possible: (i) the enzyme diffuses inside the nanochannels, converts the substrate, the product leaves the nanochannels; (ii) the substrate diffuses from the nanochannels to the external medium and is then converted. The typical sizes of the enzyme (around 5 nm diameter) and the substrate (around 0.8 nm) are significantly different and should be compared to the sizes of the nanochannels. 

From the data displayed in Figure 3c,d, it appears that over the first 5 min a steep increase of both p-nitrophenyl phosphate and p-nitrophenol absorbance is observed for all systems (in the absence or the presence of increasing amounts of SS). This initial effect can be probably attributed to a part of the substrate localized at the interface of the lipid mesophase and the surrounding medium, which freely diffuses in the buffer. There, AP readily converts it to p-nitrophenol.

Conversely, for longer times, the increase in both p-nitrophenyl phosphate and p-nitrophenol absorbance over time is slower, consistent with the conversion of the substrate confined in the aqueous channels.

For all the investigated liquid crystalline scaffolds, the size of p-nitrophenyl phosphate is smaller than the size of the water nanochannels. Therefore, we can expect an efficient encapsulation within the mesophase. However, the substrate diffusion towards the surrounding medium (driven by a chemical potential imbalance), can critically depend on the size of the mesophase nanochannels, which vary as a function of SS amounts. As we can see from Figure 3c, the kinetic profile at each SS concentration, shows that the reaction proceeds slowly for Phyt/H_2_O and that the conversion rate gradually increases with addition of sucrose stearate. We can correlate these experimental results to the SAXS data, which account for a swelling of the nanochannels as SS% is increased. This is not surprisingly, since several reports in the literature show that the diffusion rate of molecular probes depend on the channel size [8,35,36]. Therefore, in this second reaction regime the diffusion of the substrate from the nanochannels is the rate determining step.

Taking into account the displayed experimental data, and considering the different sizes of the enzyme and of the substrate also in comparison with the nanochannels diameters, we can conclude that the most probable mechanism for the enzymatic reaction, with the substrate confined in the aqueous channels, is a release of the substrate (first from the surface of the mesophase, then from the aqueous nanochannels) to the aqueous environment, followed by the conversion of the substrate into the reaction product. On the contrary, the enzyme diffusion inside the nanochannels, to convert the substrate, is probably hampered by the larger enzyme size in comparison to the channel’ size.

We should point out that, if the whole reaction kinetics is considered, even if the amount of p-nitrophenyl phosphate released and converted by the enzyme is higher for the primitive cubic structure, the final absorbance value is significantly different from the theoretical value expected considering a complete release of substrate, even after six days of reaction. The origin of this inconsistency could be related to two possible reasons: a first possibility is that, due to the domains of liquid crystals not interconnected to each other and separated by grain boundaries, a complete release of the substrate could not be observed [40,41]. A second possible explanation is that the AP enzymatic function is inhibited by the presence of high concentrations of the enzymatic product (p-nitrophenol). This latter hypothesis is in agreement with the release profile of the substrate followed for long times at 310 nm: as a matter of fact, it appears that the substrate concentration in solution keeps increasing (due to its continuous release from the cubic phase), without being mirrored by the same trend of the product (monitored at 400 nm). However, for relatively short times the rate-determining step of the reaction kinetic can be attributed to the release of the substrate to the reaction environment, while its conversion is a relatively fast process. 

In general, all the initial velocities of substrate conversion are decreased by two orders of magnitude when the substrate is confined into the mesophases with respect to the substrate freely diffusing in buffer solution (See Table 2). This diffusion rate is controlled, with a good approximation, by the size of the water channels, which is dependent on the SS amount: the Phyt/SS/H_2_O mixed mesophases can, therefore, be of interest in the biomedical field as drug delivery vehicles to release continuously active molecules in a structurally controlled manner. As an example, while binary mixture Phyt/water present an initial velocity of the product formation of around 3.78 × 10^−4^ mmol/min, and the maximum UV absorbance reached after three days, in case of the ternary matrix the release can be accelerated to an initial velocity of 8.56 × 10^−4^ mmol/min and a maximum absorbance reached in two days with the higher amount of SS tested. 

Moreover, the initial velocity, after the steep increase, varies gradually increasing the dimension of the nanochannels: the inclusion and the release of small active molecules from the liquid crystals can be finely tuned with the chemical composition in order to obtain the best condition needed for the real application.

### 2.4. Alkaline Phosphatase Inclusion in Lipid Mesophases

As a final experiment we investigated the inclusion of the enzyme AP, in Phyt/H_2_O mesophase in the absence and the presence of SS, to monitor the effect of enzyme confinement on the enzymatic reaction. As previously stated concerning the substrate confinement, also the inclusion of the enzyme in the liquid crystalline mesophases did not significantly affect the structure of the mesophases, as highlighted from SAXS (see SI for details).

**Figure 4:**
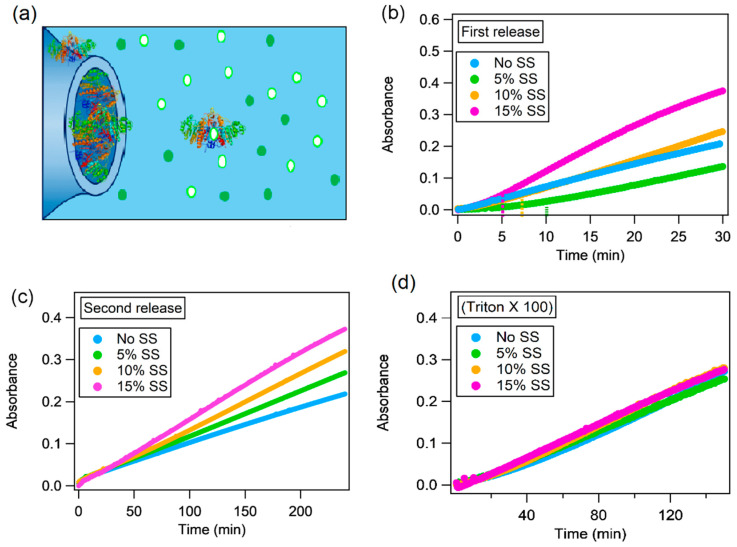
Reaction kinetics with the enzyme confined in liquid crystalline mesophases. (**a**) Sketch of the experiment of AP-catalysed reaction with the enzyme confined in the cubic mesophase; (**b–d**) UV–vis absorbance of p-nitrophenol in solution, recorder at 400 nm for the initial 30 min (**b**), for additional 250 min (upon buffer replacement with a fresh buffer and substrate solution, as described in the text) (**c**), and, finally, upon disruption of the mesophase with Triton-X-100 (**d**), as described in the text. The different curves are related to experiments performed with the enzyme confined in the different mesophases: No SS (cyan markers), 5% SS (green markers), 10% SS (orange markers) and 15% (purple markers).

To monitor the effect of enzymatic confinement on the AP enzymatic reaction, we adopted a similar experimental set-up as that described in the previous paragraph, with a different localization of the enzyme and the substrate, i.e., the dry films of Phyt and Phyt/SS were hydrated with an aqueous solution of AP and then they were immersed in a solution of the substrate. The enzymatic reaction was followed for neat Phyt/H_2_O and swelled (Phyt/H_2_O additioned with SS) cubic phases in a buffer solution containing 6 × 10^−5^ M of the substrate. First, we monitored the enzymatic reaction at 400 nm during the first 90 min of contact of the mesophase with the substrate containing buffer (Figure 4b). In this way, the release of some non-encapsulated enzyme and/or the enzyme localized quite close to the cubic phase/aqueous environment interface, was monitored.

As a second step, the cubic mesophase was placed in another cuvette, with fresh substrate at the same concentration as the first solution. In this way, we monitored the enzymatic catalysis due to AP encapsulated within the cubic phase (Figure 4c). 

Considering the first experiment, i.e., the cubic phase with the encapsulated enzyme directly immersed in the substrate solution, we notice a different mechanism in the presence of sucrose stearate. In particular, for all mesophases containing SS, two trends can be identified in the kinetic profiles. To interpret this behaviour, in the case of the Phyt/water sample, we should consider that part of the enzyme can be localized at the liquid crystalline phase/water interface and it is quickly released. At this point, the AP in solution is available to convert the substrate molecules, while the remaining AP resides inside the liquid crystalline structure catalysing the substrate conversion more slowly. In line with this hypothesis, samples with varied SS amount exhibit a lag time [42], between the first and second enzymatic kinetic trend. This is progressively decreased, enhancing the SS concentration: about 10 min for 5% *w*/*w* SS, 7–8 min in the case of 10% *w*/*w* SS and 5 min with 15% *w*/*w* SS (Figure 4b). Considering the swelling of the mesophase induced by the addition of SS, we can relate this variable effect to a variable amount of the enzyme trapped in the mesophases due to the higher water fraction characterizing the mesophase. Indeed, for the binary system, i.e., Phyt/H_2_O in the absence of SS, the trend of the kinetic curve is monotonous (Figure 4b), suggesting that, in this case, the encapsulation of the enzyme in the liquid crystalline phase is strongly hampered, due to the small size of the nanochannels. 

After the first trend, the second part of the curve proceeds with a faster rate for the systems with higher SS percentage, suggesting that the size of the nanochannels has a prominent role. 

After this first step, we performed a control experiment (Figure 4d) to verify that: (i) a significant AP amount was still present inside the mesophases after the first release; (ii) the AP amount was comparable for the mesophases, differing by SS amount. To this purpose, a set of “control” mesophases, after the first release step, was disassembled by immersion in a 24 mg/mL solution of Triton X 100. The non-ionic surfactant, at relatively high concentration [43,44] is able to disrupt the lipid bilayer to yield mixed micelles, with simultaneous quantitative release of AP originally contained in the liquid crystalline mesophase. The AP amount was measured in terms of enzymatic activity. The substantial overlap of the kinetic profiles (Figure 4d) confirms that a significant amount of AP, practically identical for all samples, is still present in the mesophases after the first release. 

Figure 4c reports the kinetic profiles concerning the second step, i.e., the immersion of the mesophases in fresh substrate solutions. Not surprisingly, the conversion rate is strictly dependent on the size of the nanochannels.

Similarly to Section 2.3, it is possible to speculate on the possible pathways for the enzymatic reaction to proceed, considering that at time zero AP enzyme is confined in the liquid crystalline mesophase and the substrate is dispersed in the surrounding medium. As previously mentioned, the enzyme is M_W_ = 140 kDa, corresponding to dimensions of around 5 nm [45]; considering a partial distribution of the enzyme between the lipid leaflet and the nanochannels of the cubic mesophases, the encapsulation of the protein could be achieved. However, considering the structure of the aqueous regions as a series of necks and cages, we can probably expect that, in the absence of a chemical potential driving its diffusion from the mesophase to the surrounding medium, the enzyme will be more conveniently localized in the cages of the structure, rather than in the nanochannels. The kinetic behaviour might be related either to the release of the encapsulated enzyme to the aqueous environment (whose rate can increase due to the swelled nanochannels), or to the penetration of the substrate inside the nanochannels of the liquid crystalline mesophase (which, similarly, increases with the nanochannel size), where it meets the enzyme, and subsequently it is released as a reaction product. The ensemble of experimental data does not allow discriminating which pathway is occurring, whether the substrate diffuses in the mesophase or the enzyme is released. A simple size argument would rule out AP diffusion from the mesophase to the external medium as the prevailing mechanism, but we are not currently able to rule out the simultaneous substrate diffusion in the mesophase. As mentioned above, the presence of additives results in the swelling of the mesophase, thus for the higher SS amount both the mechanisms are possible, and the results may be a combination of both the pathways. However, with the lower SS concentration, the enzyme release is less feasible, favouring the diffusion of the substrate into the channels. 

**Figure 5:**
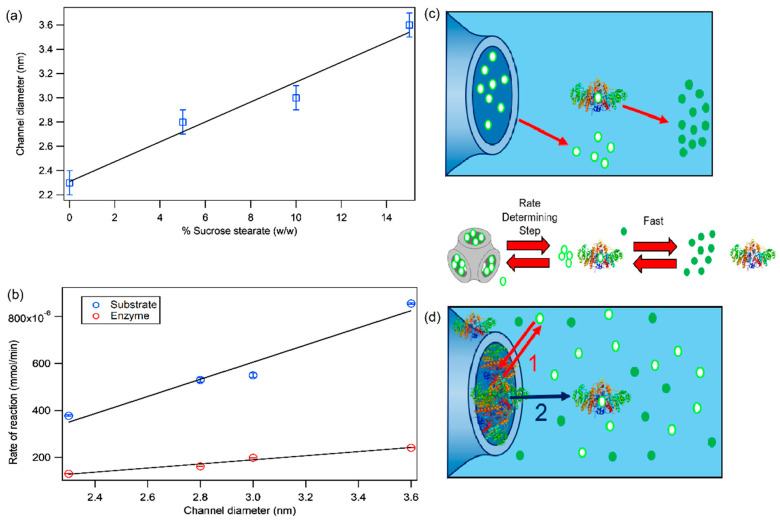
Summary: effect of substrate and enzyme confinement on AP-catalysed reaction rate and mechanism. (**a**) Channel sizes of Phyt cubic mesophases as a function of the sucrose stearate content (%) (blue markers); linear fit of the experimental data (black continuous line); (**b**) Experimental rates of the enzymatic reaction, vs. channels size (nm), for the case of confined substrate (blue markers) and confined enzyme (red markers); linear fit of the experimental data (black continuous lines); (**c–d**) scheme sketching the hypothesized reaction mechanisms in the case of the confined substrate (**c**) and confined enzyme (**d**), according to the experimental data: in the case of the confined substrate the hypothesized mechanism is the diffusion of the substrate from the aqueous channels to the surrounding solution as the limiting reaction step, followed by the fast conversion into the product by the enzyme (see scheme d); in the case of the confined enzyme, two possible mechanisms could be considered, or a combination of both, i.e., the escape of the enzyme in the surrounding solution where the substrate is dispersed ready to be converted (mechanism 2, black arrow), or else the substrate diffuses in the aqueous channels, where the enzyme is confined, and it is converted into the product, which then diffuses back in the surrounding solution (mechanism 1, red arrows).

Figure 5 summarizes the main results of the study, putting in relationship the structural features of mixed Phyt/SS/H_2_O mesophases with the reaction rate of p-nitrophenylphosphate conversion into p-nitrophenol, upon confinement of the substrate and of the enzyme in the aqueous channels of the liquid crystalline scaffolds. 

Figure 5a reports the diameter of the aqueous channels of Phyt/SS liquid crystalline mesophases as a function of the amount of added SS. The graph clearly highlights that, as discussed in Section 2.1, the amount of SS added to Phyt mesophase is an effective control parameter to finely tune the size of the aqueous channels of the liquid crystalline mesophases, to host/confine chemical species of different sizes. 

Figure 5b (blue markers) reports the reaction rates of p-nitrophenylphosphate conversion, upon confinement of the substrate in Phyt/SS mesophases, as a function of the diameter of the aqueous channels, with increasing SS%. The mechanism of the enzymatic reaction, which has been discussed in Section 2.3, is then sketched in Figure 5c: the rate-determining step of the reaction is the diffusion of the substrate from the aqueous channels of the liquid crystalline mesophase to the surrounding solution, where a fast conversion of it into the product occurs, catalysed by the enzyme. The ratio between the size of the substrate and the diameter of the aqueous channels controls the diffusion rate and, therefore, the rate of the overall enzymatic reaction. As a matter of fact, as reported in Figure 5b, the reaction rate increases almost linearly with the increase of the channels size. The red markers in Figure 5b are related to confinement of the enzyme in Phyt/SS mesophases. In this case, as sketched in Figure 5d, the mechanism of the enzymatic reaction is not unambiguously determined: as previously discussed, it might be either the enzyme escaping from the aqueous channels to the surrounding solution where the substrate is dissolved (mechanism 2), or the substrate, diffusing into the aqueous channels of the lipid mesophase, to interact with the enzyme, be converted in the reaction product and subsequently diffuse back in solution (mechanism 1), or a combination of both, depending on the swelling degree of the aqueous channels. All three hypotheses are consistent with the highlighted strong decrease in all reaction rates, as compared to the case of the confined substrate (see Figure 5b red markers compared to the blue markers), attributable to the complex, multistep diffusion process involving small molecules (the substrate and the product), or to the diffusion of the bulky enzyme, characterized by significant confinement effects. In addition, also in the case of confined enzyme, as for the confined substrate, the increase of the reaction rates with the increase of the aqueous channels sizes appears almost linear. 

Overall, the data here discussed highlight that the rate and mechanism of the enzymatic reaction depend on the structure of the lipid mesophase, in particular on the size of the aqueous channels, which is finely tunable by controlling the additive concentration. 

## 3. Materials and Methods

### 3.1. Materials

Phytantriol (Phyt) was a gift of Royal DSM (purity >99%). Sucrose stearate (SS) (Crodesta F110-PW-(RB)) was a gift of Croda Europe Ltd. Rawcliff Bridge, Goole, United Kingdom. TRIS buffer was prepared with Trizma^®^ hydrochloride (>99.0%), TRIZMA^®^ BASE (>99.9%) that were both purchased from Fluka-Sigma Aldrich (St. Louis, MO, USA). The enzyme alkaline phosphatase (AP) (REF 11 097 075 001) 1000 U/mL purchased from Roche Diagnostic (Indianapolis, USA), the substrate p-nitrophenyl phosphate (>99% purity) was purchased from Fluka-Sigma Aldrich (St. Louis, MO, USA). The same for Triton X 100 (>99.0% purity).

### 3.2. Instruments

#### 3.2.1. Small Angle X-ray Scattering

SAXS measurements were performed on a S3-MICRO SAXS/WAXS instrument (HECUS GmbH, Graz, Austria) which consists of a GeniX microfocus X-ray sealed Cu Kα source (Xenocs, Grenoble, France) with power of 50 W. The source provides a focused X-ray beam with k = 0.1542 nm Cu K**α** line. The instrument is equipped with two one-dimensional (1D) position sensitive detectors, (HECUS 1D-PSD-50M system) each detector is 50 mm long (spatial resolution 54 nm/channel, 1024 channels) and covers a q-range of 0.003 < q < 0.6 Å-1 (SAXS) and 1.2 < q < 1.9 Å-1 (WAXS). The temperature was controlled by means of a Peltier TCCS-3 Hecus. SAXS curves of bulk cubic phases were recorded at 25-30-35-40-45-50 °C in a kapton solid sample holder. SAXS profiles were then recorded for the different samples 15 min each temperature, waiting 20 min for thermal equilibration. 

#### 3.2.2. UV–Vis Spectrophotometer: 

UV–vis measurements were performed on UV-Cary 3500 Agilent Technologies, which allows data collection in the range 190–1100 nm wavelength with different available bandwidths that can be selected between 0.1 and 5.0 nm at 0.01 nm intervals: the light spot on the sample is 1.5 mm. The instrument presents a Xenon flash lamp, double out-of-plane Littrow monochromator for fast data collection. There are eight cuvette position with eight detectors collecting the light signal which arrive on the sample at 1.5 cm from the bottom of the cuvette. It presents a magnetic stirrer able to mix the solution in cuvette with the appropriate stir bar. The temperature is controlled by different Peltier blocks in the range 0–100 °C.

### 3.3. Samples Preparation

#### 3.3.1. SAXS Bulk Cubic Phases

They were prepared in the presence or in the absence of the additive sucrose stearate according to the following procedure: 30 mg of Phyt were weighted in a 2 mL glass flask in the absence (for pure Phyt systems) or in the presence of appropriate amounts of sucrose stearate in order to reach a final amount in the final lipid mixture of 5, 10, or 15% wt. About 0.5 mL of chloroform was used to solubilize the mixtures, then the solvent was removed under a gentle nitrogen flux. The lipid films were left under vacuum overnight, then hydrated with 16 **μ**L Milli-Q water and left for at least 12 h to equilibrate before the experiments.

#### 3.3.2. UV–Vis Bulk Cubic Phases

For the UV–vis experiments, the same protocol described for SAXS was employed for the mesophases preparation, with doubled lipid amount. The cubic phases prepared for the experiment of p-nitrophenyl phosphate encapsulation were prepared by hydrating the lipid films with 31 µL of TRIS buffer solution pH 7.5 containing 5 × 10^−3^ M of the substrate, in order to reach a theoretical final concentration in the cuvette of 6 × 10^−5^ M. The cubic phases prepared for the experiment of AP encapsulation were prepared by hydrating the lipid films with TRIS buffer pH 7.5 solution containing 16 U/mL AP enzyme, with a theoretical final value in cuvette after dilution (final volume 2.5 mL) of 0.2 U/mL. To perform these measurements, we built-up a home-made modified plastic flask where a plastic ring was tied to the top of cuvette leaving the mesophase infusing into the buffer until the reaction was complete. The experimental set-up improved the diffusion of the confined substrate or enzyme from the cubic mesophase to the bulk solution, avoiding the mesophase deposition at the bottom of the cuvette. Moreover, in order to homogenize the reaction solution, the buffer was stirred with cuvette stirrer bars during all the time of the experiments.

## 4. Conclusions

This contribution reports on the enzymatic kinetics of the conversion of p-nitrophenyl phosphate in p-nitrophenol, catalysed by alkaline phosphatase, upon confinement either of the enzyme or of the substrate in phytantriol liquid crystalline mesophases, as the structural parameters are varied.

A library of phytantriol liquid crystalline mesophases with variable and controlled structural parameters is achieved through the inclusion of selected amounts of the additive sucrose stearate (SS). The resulting phytantriol/sucrose stearate mixed mesophases are characterized by a tunable structure, both concerning the structural arrangement and the diameter of the water nanochannels.

The UV–vis spectroscopy investigation on the enzymatic reaction highlighted that the alternative inclusion of either the substrate or the enzyme in the cubic scaffold, has different impacts on the reaction kinetics, which is deeply affected by the structure of the mesophase.

Specifically, the confinement of the substrate or of the enzyme in the mesophase results in a significant slowing down of the reaction velocity, whose extent critically depends on the ratio between the size of the confined species (a small molecule in the case of the substrate, a macromolecule in the case of the enzyme) and the nanochannels diameter.

In summary, the structural control on the self-assembly of the mesophases can be suitably exploited to control the loading and release profile of drugs of different sizes (a small molecule or an enzyme) and even a reaction kinetics. These systems, i.e., Phyt/SS/H_2_O ternary mesophases, characterized by a variable and controlled structure, which is closely related to the loading-release properties of the mesophase, represent thus a suitable scaffold for the confinement and sustained release of active principles of different sizes.

## Figures and Tables

**Table 1:** Lattice parameter d (nm) of Phyt mesophases doped with different concentrations (0%, 5% *w*/*w*, 10% *w*/*w*, 15% *w*/*w*) of SS in excess water, measured at different temperatures in the range T = 25–50 °C.

	0% SS		5% SS		10% SS		15% SS	
T (°C)	d (nm) ^4^	dw (nm)	d (nm)	dw (nm)	d (nm)	dw (nm)	d (nm)	dw (nm)
**25**	6.6 ^1^ ± 0.1	2.3 ± 0.1	7.2 ^1^ ± 0.1	2.8 ± 0.1	7.5 ^1^ ± 0.1	3.0 ± 0.1	10.5 ^3^ ± 0.2	3.6 ± 0.1
**30**	6.5 ^1^ ± 0.1	2.2 ± 0.1	7.0 ^1^ ± 0.1	2.6 ± 0.1	7.2 ^1^ ± 0.2	2.8 ± 0.1	10.3 ^3^ ± 0.1	3.4 ± 0.1
**35**	6.4 ^1^ ± 0.1	2.2 ± 0.1	6.7 ^1^ ± 0.1	2.4 ± 0.1	7.1 ^1^ ± 0.2	2.7 ± 0.1	9.9 ^3^ ± 0.2	3.2 ± 0.1
**40**	6.3 ^1^ ± 0.1	2.0 ± 0.1	6.6 ^1^ ± 0.1	2.3 ± 0.1	6.9 ^1^ ± 0.1	2.6 ± 0.1	7.4 ^1^ ± 0.2	2.9 ± 0.1
**45**	4.8 ^2^ ± 0.2	1.9 ± 0.1	6.6 ^1^ ± 0.2	2.3 ± 0.1	6.7 ^1^ ± 0.2	2.4 ± 0.1	7.1 ^1^ ± 0.1	2.7 ± 0.1
**50**	4.0 ^2^ ± 0.1	1.2 ± 0.1	6.4 ^1^ ± 0.2	2.2 ± 0.1	6.6 ^1^ ± 0.1	2.3 ± 0.1	7.0 ^1^ ± 0.1	2.6 ± 0.1

^1^ Pn3m structure; ^2^ H_II_ structure; ^3^ Im3m structure; ^4^ Phyt/water lattice parameters reported from reference [37].

**Table 2:** Enzymatic reaction rates expressed in mmol/min of converted substrate for the free substrate (No mesophase) and for the substrate confined in mesophases with increasing amounts of the additive SS.

Sample	Rate (mmol/min) Confined Substrate
**No mesophase**	1.18 × 10^−2^ (±6 × 10^−5^)
**Phyt**	3.78 × 10^−4^ (±3 × 10^−6^)
**Phyt/5% SS**	5.30 × 10^−4^ (±1 × 10^−5^)
**Phyt/10% SS**	5.5 × 10^−4^ (±1 × 10^−5^)
**Phyt/15% SS**	8.56 × 10^−4^ (±3 × 10^−6^)

## Supplementary Materials

Supplementary materials can be found at https://www.mdpi.com/1422-0067/21/14/5116/s1.

## Abbreviations

AP	Alkaline Phosphatase
Phyt	Phytantriol
SS	Sucrose Stearate
GMO	Glycerol monooleate
IPMS	Infinite Periodical Minimal Surfaces
SAXS	Small-Angle X-ray Scattering
