# Supplementary material for: Controlling the Kinetics of an Enzymatic Reaction through Enzyme or Substrate Confinement into Lipid Mesophases with Tunable Structural Parameters
Source: Int J Mol Sci. 2020 Jul 20;21(14):5116. doi: 10.3390/ijms21145116 (PMC7404178; doi:10.3390/ijms21145116)
Supplement: Supplementary file 1 [file ijms-21-05116-s001.pdf]

Supporting Information for:

Controlling the kinetics of an enzymatic reaction through enzyme or substrate confinement into lipid mesophases with tunable structural parameters

Marco Mendozza[1], Arianna Balestri[1], Costanza Montis[1,*] and Debora Berti[1]

[1] Department of Chemistry "Ugo Schiff" and CSGI, Via della Lastruccia 3-13, 50019, Florence, Italy;
[*] Correspondence: costanza.montis@unifi.it; Tel.: +39 055 457 3017

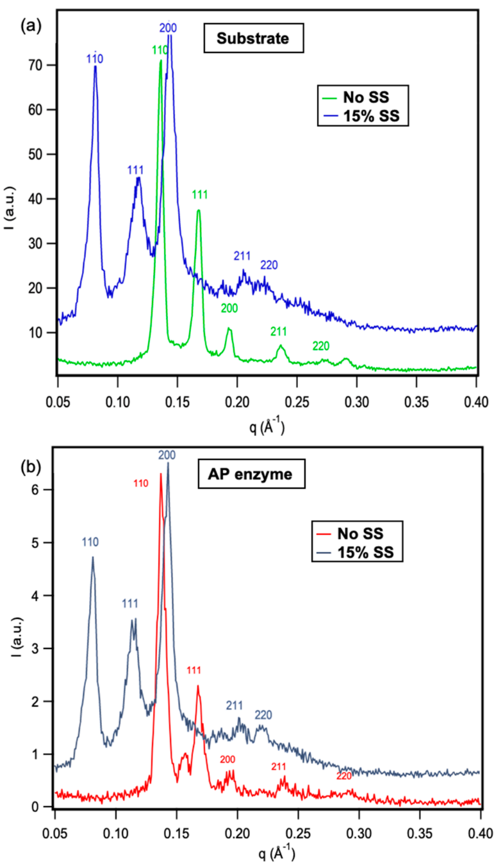

Figure S1. **Structure of Phyt mesophases doped with substrate and AP enzyme.** SAXS profiles of Phyt mesophases containing substrate (a) and AP enzyme (b), in excess water, measured at 25°C with and without the Sucrose Stearate (15%SS and No SS).
